# Anticancer effect of *Illicium verum* (star anise fruit) against human breast cancer MCF-7 cell line

**DOI:** 10.12669/pjms.39.1.6580

**Published:** 2023

**Authors:** Asra Khan Pahore, Shagufta Khan, Nasim Karim

**Affiliations:** 1 Dr. Asra Khan Pahore, BDS, MPhil. Lecturer, Department of Pharmacology, Altamash Institute of Dental Medicine, Karachi, Pakistan; 2 Dr. Shagufta Khan, MPhil, PhD. Assistant Professor, Department of Biological & Biomedical Sciences, Aga Khan University, Karachi, Pakistan; 3 Prof. Dr. Nasim Karim, MBBS, MPhil, Ph.D., Post-Doc. Head Department of Pharmacology, Bahria University Medical & Dental College, Sailors Street, Adjacent PNS Shifa, DHA Phase-II, Karachi, Pakistan

**Keywords:** Apoptosis, Breast neoplasms, Cell line, Cell proliferation, Illicium, MCF-7 cells

## Abstract

**Objective:**

To investigate the anticancer effect of *Illicium verum* against human breast cancer MCF-7 cell line.

**Methods:**

An experimental study was conducted in Multidisciplinary and Tissue Culture Laboratory, Aga Khan University in collaboration with Pharmacology Department of Bahria University Medical and Dental College, Karachi, Pakistan from January 2021 to June 2021. MCF-7 cells of Luminal-A breast cancer were seeded in 96-well plate and treated with *I.verum* methanol extract. After incubation, MTT (3-(4,5-dimethylthiazol-2-yl)-2,5-diphenyltetrazolium bromide) dye was used for cell viability and cell proliferation assays to determine the number of dead and viable cells, and the absorbance was measured using an enzyme-linked immunosorbent assay (ELISA) plate reader. In cell viability assay, different doses of *I. verum* methanol extract were used to treat the MCF-7 (0.25, 0.5, 1, 3, 6, 12, 25, and 50μg/ml) cells. For apoptosis analysis, the cells were processed with 4´, 6-diamidino-2-phenylindole fluorescent nuclear dye (DAPI) and were examined for fluorescence intensity and apoptotic cells. For cell proliferation assay and apoptosis the IC50 dose of 5.5μg/ml *I. verum* methanol extract was used.

**Results:**

The MCF-7 cells showed a significant reduction (p-value <0.01) in cell viability in the presence of all tested doses of *I. verum* methanol extract, except for the dose of 0.25μg/ml. The IC_50_ dose 5.5μg/ml of same extract also showed a significant reduction (p-value <0.01) in cell proliferation and apoptosis induction in MCF-7 cells.

**Conclusions:**

*Illicium verum* methanol extract possesses very potent anticancer action against MCF-7 cells through cytotoxicity, reduction, and inhibition of cancer cells and by inducing apoptosis.

## INTRODUCTION

Breast cancer is the most prevalent malignant tumor and a foremost reason of death in females of all over the world. It is also the most prevailing cancer among females in Pakistan, as per age-standardized incidence rate (ASIR).^1^ In comparison to other Asian countries; Pakistan has relatively high incidence and mortality rates, as indicated that one in every nine women would experience breast cancer once in their life time.^2^

Breast cancer is classified molecularly as Luminal-A, Luminal-B, HER-2, or Triple-negative types. The MCF-7 cell line belongs to most prevalent subtype which is represented as Luminal-A; is estrogen and progesterone receptor positive, HER-2 receptor negative, and of low grade with an excellent prognosis.^3^

Currently, several breast cancer treatments are available such as surgery, radiotherapy, chemotherapy, hormonal and targeted therapy. Although these modalities are effective but they are strongly associated with negative impact on a patient’s health. On the other hand, medicinal plants have fewer adverse effects and can thus be used as a preferred alternative treatment for cancer.^4^

A medicinal plant is one which possesses few active compounds with biological action(s). Plant-derived traditional medicines are now favored over western modern medications for many disease management due to their superior efficacy, acceptance, accessibility, and cost-effectiveness.^5^

*Illicium verum* is middle-sized, scented, evergreen tree of the Illiciacea family. Apart from its use as a spice in cooking, *I. verum* has been used for the treatment of abdominal colic, flatulence, rheumatism, and spasmodic pain. It also has many other medical properties such as antibacterial, antiviral, antifungal, antioxidant, and anticancer properties.^6^ The anethole component of *I. verum* manifests anti-proliferative, anti-inflammatory, and pro-apoptotic effects on the MCF-7 and MDA-MB-231 cell lines.^7^ Further it has been documented that the anethole component of the *I. verum* induced apoptosis in MDA-MB-231 cell lines.^8^

As per our literature search from 2011 to 2021 in multiple search engines, no work has yet been reported on anticancer effects and mechanism of action of *I. verum* dried fruit methanol extract against human breast cancer cell lines. *I. verum* fruit extract has medicinal values and anti-cancer potential against various cancers. The current work was designed to explore the anticancer activities of methanol extract of dried fruit of *I. verum* against human breast cancer MCF-7 cell line based on the aforementioned literature knowledge.

## METHODS

An experimental cell culture study was conducted as MPhil research in Multidisciplinary and Tissue Culture Laboratory, Department of Biological and Biomedical Sciences, Aga Khan University in collaboration with Pharmacology Department of Bahria University Medical and Dental College (BUMDC), Karachi, Pakistan from January 2021 to June 2021 after receiving approval from Institutional Ethical Review Committee (Reference No. BUMDC-ERC 11/2021, Dated: 15-Jan-21). The extract was prepared by purchasing dried fruit of *I. verum* (5kg) from a local market. A voucher specimen (No IV-F-27-12-120) was deposited in the herbarium, Department of Biological and Biomedical Sciences, AKUH. Fruit of *I. verum* was soaked in 5L of aqueous methanol 30:70 (1.5L and 3.5L) for three days at room temperature. The 1^st^ filtrate was obtained using initially a muslin cloth and then a Whatman filter paper. The marc left was again soaked in the same ratio for three days each at room temperature to get the 2^nd^ and 3^rd^ filtrate. The 1^st^, 2^nd,^ and 3^rd^ filtrates were combined and the solvent was evaporated via a rotary evaporator (Buchimodel R -210 Switzerland) to obtain methanol extract of *I. verum* (IV- extract 1599g). The extract was stored at 4 ºC and used for cell culture studies.^9^

Human breast cancer cell line MCF-7 (ATCC, Manassas, VA, USA) was grown in Dulbecco’s modified Eagle medium (DMEM) added with 10% fetal bovine serum (FBS) and 1% antibiotic-antimycotic (ABAM) solution.

In cell viability assay, the MCF-7 cells were seeded at a density of 1 × 10^4^/well in a 96-well cell culture plate. After 24hrs of incubation, MCF-7 cells were treated with various concentrations of prepared of *I. verum* extract (0.25, 0.5, 1, 3, 6, 12, 25, 50μg/ml) for 48hrs. After incubation, the respective culture medium was replaced with fresh media containing 3-(4,5-dimethylthiazol-2-yl)-2,5-diphenyltetrazolium bromide (MTT, 0.5mg/mL), then incubated for 4hrs at 37°C in a humidified 5% CO2 incubator. To evaluate cell viability, the formazan crystals were dissolved in Dimethyl sulfoxide (DMSO) and absorbance was read using the enzyme-linked immunosorbent assay (ELISA) plate reader (BIO-RAD iMark™ Model168-1135 USA) at 550nm. To calculate the percent of dead cells and viable cells following formula was used.^10^

% of cell viability = [(A test – A blank) / (A control – A blank)] ×100 Where = **A:** absorbance

In cell proliferation assay, the MCF-7 cells were seeded at a density of 1 × 10^3^/well in a 96-well plate. After 24hrs of incubation, these cells were treated with IC_50_ dose of *I. verum* extract (obtained from cell viability assay) for 1-3-days. At the end of indicated time points, following living cells were calculated as described above. The cell growth curve was plotted by live cells in 1-3-days (i.e. 24hrs, 48hrs, and 72hrs) time period.^10^

Analysis of apoptosis induction via DAPI staining was performed as described earlier with slight modification.^11^ The 1 × 10^4^ MCF-7 cells were seeded in 100μl DMEM in each well of the 96-well culture plate for 24h at 37ºC and 5% CO2 in humidified air. These cells were then treated with IC_50_ dose (obtained from MTT assay) of *I. verum* extract for 48h. After treatment with extract, media was removed. These cells were washed with phosphate buffer saline (PBS) and fixed in 4% paraformaldehyde for ten minutes. Later, the cells were permeabilized with buffer (0.5% Triton X-100 and 4% paraformaldehyde) and stained with 50μl of 4´, 6-diamidino-2-phenylindole fluorescent nuclear dye (DAPI) with the last concentration of 1μg/ml. After 1h, the cells were observed for the fluorescence intensity and apoptotic cells. The images were taken and cells were counted by using a fluorescent microscope. The percent apoptotic cell was calculated by using following formula.^12^

% of apoptotic cell = (control cells - apoptotic cells) × 100

### Statistical analysis:

Each data was presented as the mean ± standard error of mean. Statistical significance is determined by ANOVA (one-way analysis of variance) followed by Dunnett’s multiple comparison test by Graph Pad Prism 4. A p-value < 0.05 was considered statistically significant.

## RESULTS

**Fig.1:**
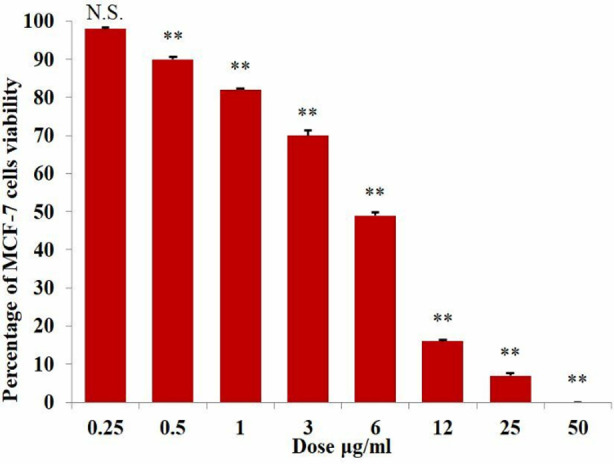
Effect of different doses of *I. verum* extract on the percentage of MCF-7 cell viability. Each bar is the mean ± SEM of 3 determinations, each in triplicate. Asterisks indicate significant inhibition of cell viability as compared to control. The non-significant difference represented by N.S. using ANOVA. Whereas, results obtained from the Dunnett multiple comparison tests also showed a significant difference between the percentages of cell viability inhibitory effects of each dose (i.e., each bar).

When MCF-7 cells were treated with *I. verum* extract at 0.25μg/ml dose, after 48hrs of incubation no significant reduction was observed in cell viability. The *I. verum* extract showed a significant dose (0.5 – 50μg/ml) dependent reduction in viability of MCF-7 cells. The extract showed 90% and complete inhibition (0%) of cell viability at 0.5μg/ml and 50μg/ml dose, respectively (Fig.1).

After 48hrs of incubation at the lowest tested dose (0.25μg/ml) of *I. verum* extract, all MCF-7 cells were found to be alive. However, the *I. verum* extract showed a significant dose-dependent increase in cell death in the presence of 0.5 - 50μg/ml dose. The extract exhibited only 10% cell death and reached 100% of cell death at the dose of 0.5μg/ml and 50μg/ml, respectively (Table-I).

**Table I:** Effect of different doses of *I. verum* extract on the percentage of MCF-7 cell death.

Dose μg/ml	Mean	Standard deviation	Standard Error	p-value
0.25	2.333	0.577	0.333	>0.05^N.S.^
0.5	10.000	1.000	0.577	<0.01
1	18.333	0.577	0.333	<0.01
3	30.333	2.309	1.333	<0.01
6	51.333	1.528	0.882	<0.01
12	84.333	0.577	0.333	<0.01
25	92.667	1.155	0.667	<0.01
50	100.000	0.000	0.000	<0.01

Each value is the mean of 3 determinations, each in triplicate; while N.S. represented non-significant cell death. The Dunnett multiple comparison tests showed a significant difference between the percentages of cell death of all the tested doses (0.5 - 50μg/ml).

**Fig.2:**
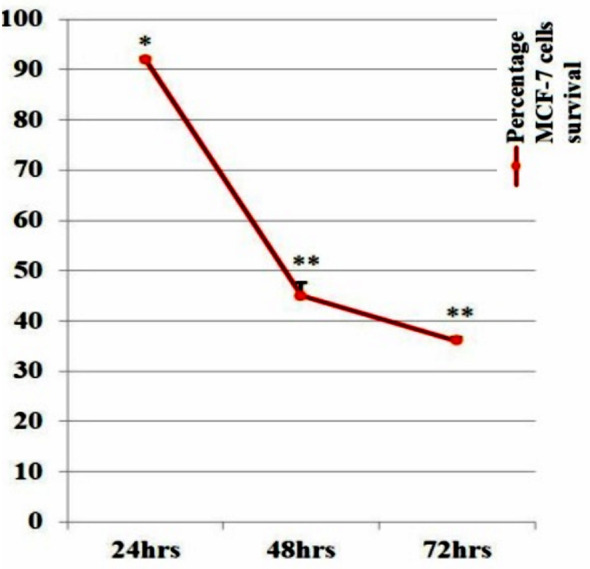
Percentage of MCF-7 cells survival during 1-3-days in the presence of IC50 (5.5μg/ml) value of *I. verum* extract. Each point is the mean ± SEM of 3 determinations, each in triplicate. Asterisks indicate significant inhibition of cells survival in comparison to respective control using ANOVA. Results obtained from the Dunnett multiple comparison test also showed a significant difference between the reduction in cells proliferation during a 1-3-days treatment period (i.e. each point).

When MCF-7 cells were treated with IC_50_ dose of *I. verum* extract obtained from MTT assay, for a period of 24hrs, 48hrs, and 72hrs. After the aforementioned incubation period, the extract showed a significant reduction in MCF-7 cells growth (proliferation) as compared to respective control (untreated cell growth). The MCF-7 cells growth was found to be 92.3%, 45.3%, and 36.3% at 24hrs, 48hrs, and 72hrs, respectively (Fig.2).

When MCF-7 cells were treated with IC_50_ dose of *I. verum* extract obtained from MTT assay, for a period of 24hrs, 48hrs, and 72hrs. After the aforementioned incubation period, extract showed a significant increase in MCF-7 cells death as compared to respective control (untreated cell growth). The MCF-7 cells death was found to be 8%, 51.3%, and 63.7% at 24hrs, 48hrs, and 72hrs, respectively (Table-II).

**Table II:** Percentage of MCF-7 cells death during 1-3-days in the presence of IC_50_ value of *I. verum* extract.

Dose μg/ml	Days (hours)	Mean	Standard deviation	Standard error	p-value
	1 (24hrs)	7.667	0.577	0.333	<0.01
5.5	2 (48hrs)	51.333	1.155	0.667	<0.01
	3 (72hrs)	63.667	1.155	0.667	<0.01

Each value is the mean of 3 determinations, each in triplicate. Data obtained from the Dunnett multiple comparison tests also showed a significant difference in percentage of cell death during the treatment period of 1-3-days (i.e. each day).

The treatment of MCF-7 cells at IC_50_ dose for a period of 48hrs showed condensation and fragmentation of the nuclei identified by DAPI via fluorescence microscopy. While the control (untreated) cells showed original morphology with several nucleoli. The apoptotic cells percentage was 47.7 ± 2.02. Three experiments were performed and each experiment was conducted in triplicate.

## DISCUSSION

Many cell viability studies using MTT assay provided evidence that anticancer properties reside in various plants such as the IC_50_ value of *Elaeis guineensis*, *Carthamus tenuis, Hyptis pectinata* and *Epilobium parviflorum* is 15μg/ml, 25μg/ml, 46μg/ml, 73μg/ml, respectively.^13^-^16^ Likewise, in the present investigation, IC_50_ value of *I. verum* extract was found to be 5.5 ± 0.9μg/ml which caused significant reduction in MCF-7 cells viability, indicating its anticancer activity in a dose-dependent manner (Table-I).

The MTT assay also calculates rate of cell proliferation and reduced metabolic activity which results in apoptosis that signifies reduced cell viability.^17^ It has been reported that after an incubation period of 72hrs, methanolic extract of *Berberis hispanica* showed a 50% reduction in the proliferation of MCF-7 cells.^18^ One more study reported the anti-proliferative effect of *Zaleya pentandra* extract against MCF-7 cells almost 37% after 24hrs of treatment.^19^ While another study reported the anti-proliferative effect of *Allium autumnale* extract which showed 98.5%, 99.68%, and 99.77% reduction in MCF-7 cells growth after 24hrs, 48hrs, and 72hrs of treatment, respectively.^20^

Considering the excellent anticancer activity of *I. verum* extract for MCF-7 cells (Luminal-A), its anti-proliferative potential was also assessed against MCF-7 cells at the IC_50_ dose of 5.5μg/ml. Our results showed time-dependent reduction of MCF-7 cells proliferation 7.7 ± 0.3%, 51.3 ± 0.7%, 63.7 ± 0.7% at 24hrs, 48hrs, 72hrs, respectively. Thus, aforementioned results indicated that also *I. verum* extract possesses anti-proliferative activity against MCF-7 cells.

Apoptosis is also known as automatic cell death. The constant number of breast cancer cells is conserved by a balance between proliferation and apoptosis of cells. A small variation in this, can lead to loss of homeostasis and result in cancer cells growth. Therefore, cell cycle arrest and apoptosis induction remain the main method to restrain and inhibit breast cancer cells. There is an immense concern in the advancement of newer anticancer drugs inducing apoptotic activity with specificity and have few side effects.^21^ *Petasites hybridus*, *Artemisia nilagirica*, and *Zanthoxylum armatum* extracts are reported to exert anticancer action via apoptosis in MCF-7 cells, studied by DAPI dye staining assay.^22^-^24^ In the present study, *I. verum* extract also caused significant induction of apoptosis at the IC_50_ dose of 5.5μg/ml in MCF-7 cells, confirming that the cytotoxic effect of *I. verum* extract is due to automatic cell death. These findings are consistent with the results obtained from both cell viability and cell proliferation assays.

Many studies have reported on medicinal properties of *I. verum* whereas very few studies are documented on the anticancer potential of ethanol extract of *I. verum* on different type of cancers. No data has been presented before on the methanol extract of *I. verum* against breast cancer cell line. Present study has explored a new treatment option for Luminal-A type breast cancer represented by MCF-7 cell line. Since *I. verum* is a medical plant and extract obtained from it, is expected to be cost effective with few side effects if used as a drug in patients having Luminal-A breast cancer. It is an open avenue for future researchers to conduct clinical trials on such type of patients.

### Limitations of study:

Only one (MCF-7) breast cancer cell line was used due to budget constraints and non-availability of other breast cancer cell lines in Pakistan due to severe covid-19 restrictions during the study period.

## CONCLUSIONS

*Illicium verum* methanol extract possesses very potent anticancer activity against MCF-7 cells by cytotoxicity, reduction, and inhibition of these cancer cells and apoptosis. Based on findings *I. verum* is an outstanding candidate to explore and further can be introduce as a new anticancer agent for breast cancer treatment.

### Author’s Contribution:

**AKP:** Principal investigator and researcher, did data collection, literature search, and write-up of the manuscript.

**SK:** Co-supervisor of the study, analyzed and interpreted data, reviewed and proofread the final version.

**NK:** Supervisor of the study, conceptualized and designed the study, drafted the manuscript, and revised it carefully.

**SK and NK:** They both are responsible and accountable for the accuracy or integrity of the study.
